# Two successive electrocardiograms of an old male with acute myocardial infarction: What on earth was going on?

**DOI:** 10.1111/anec.12950

**Published:** 2022-04-07

**Authors:** Xiaoqing Wang, Yongjun Chen, Xiaoyu Yang, Ling Yang

**Affiliations:** ^1^ Department of Cardiology The Third Affiliated Hospital of Soochow University Changzhou City China

**Keywords:** cardiac arrest, myocardial infarction, sudden death

## Abstract

A 68‐year‐old male complained of a sudden 2‐h chest pain accompanied by dizziness and diaphoresis. His consciousness lost several times because of ventricular fibrillation attack. Emergent CAG showed proximal left anterior descending (LAD) occlusion, but two previous successive electrocardiograms established diagnoses of non‐ST‐elevation myocardial infarction (NSTEMI) and STEMI respectively, indicating that the patient had experienced acute subtotal occlusion of proximal LAD to total occlusion of the left main coronary trunk (LMT). It is vital to identify de Winter pattern associated with proximal LAD lesion in view of the potential circulatory collapse, fatal arrhythmias and sudden cardiac death from it.

## Graphical abstract

De Winter pattern, as an uncommon electrocardiogram (ECG) manifestation, occurs in patients with proximal left anterior descending (LAD) subtotal occlusion or total occlusion with well‐developed collateral circulation. Sometimes de Winter pattern could change into ST‐elevation myocardial infarction (STEMI) associated with occluded proximal LAD and even occluded left main trunk (LMT), indicating that it is of vital clinical significance.

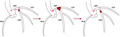

A 68‐year‐old man complained of a sudden 2‐h chest pain accompanied by dizziness and diaphoresis. The patient had a history of smoking. His consciousness lost upon arrival at the emergency room. Electrocardiogram (ECG) revealed ventricular fibrillation, which was immediately defibrillated. An urgent ECG was then performed (Figure 1), and his blood pressure was 77/51 mmHg. Ventricular fibrillation recurred a few minutes later, and another ECG was redone after defibrillation (Figure 2). The second ECG was performed 9 min after the first. The patient was immediately given aspirin (300 mg), ticagrelor (180 mg) orally, and unfractionated heparin (4000 u) intravenously. Coronary angiography (CAG) was performed an hour after the second ECG, showing total occlusion of the proximal left anterior descending (LAD) (thrombolysis in myocardial infarction [TIMI] flow = 0) without collateral circulation (Figure 3). A drug‐eluted stent was deployed in the proximal LAD, and blood flow was restored (TIMI flow = 3). Two additional cardiac arrests occurred before CAG, both dealt with defibrillation. Emergency cardiac troponin I level was 0.029 ng/ml (normal, <0.04 ng/ml) and peaked at 12.289 ng/ml after percutaneous coronary intervention (PCI). 12‐lead ECG (Figure 4) was performed on the first day after PCI. Subsequent echocardiogram showed an ejection fraction of 50%. The patient's condition was stable.

**FIGURE 1:**
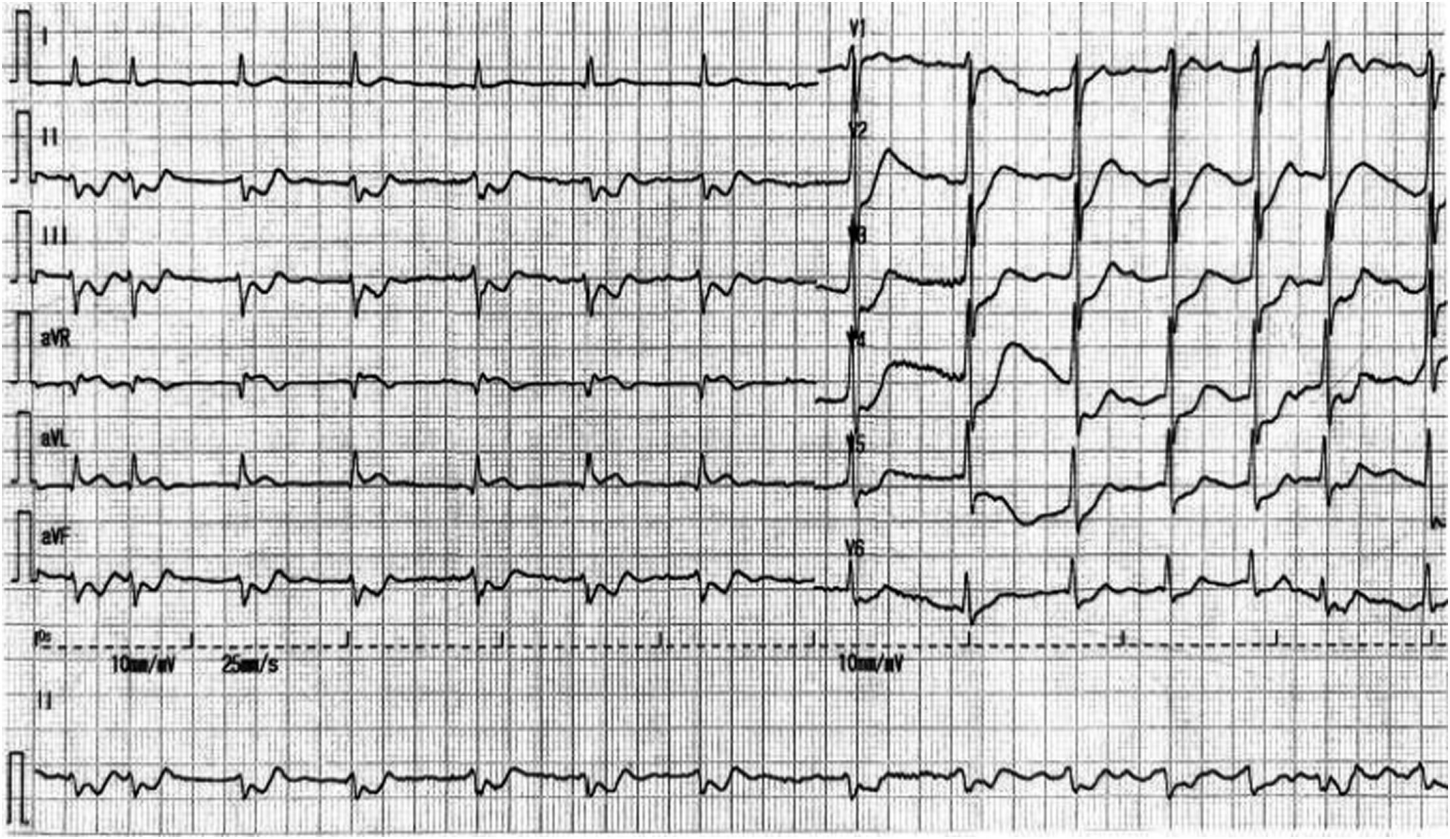
ECG performed after the first defibrillation

**FIGURE 2:**
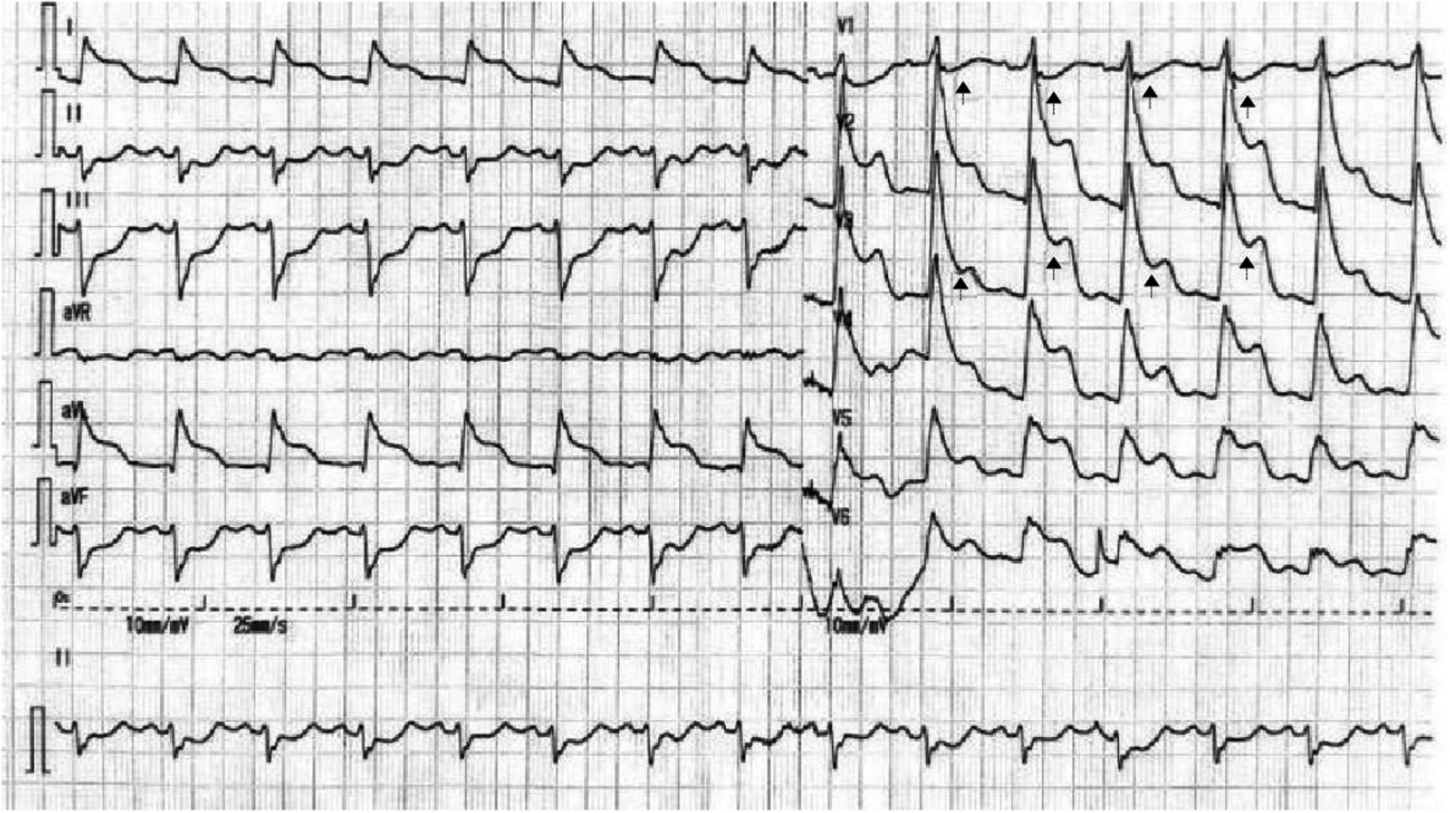
ECG was performed 9 min later than the initial ECG

**FIGURE 3:**
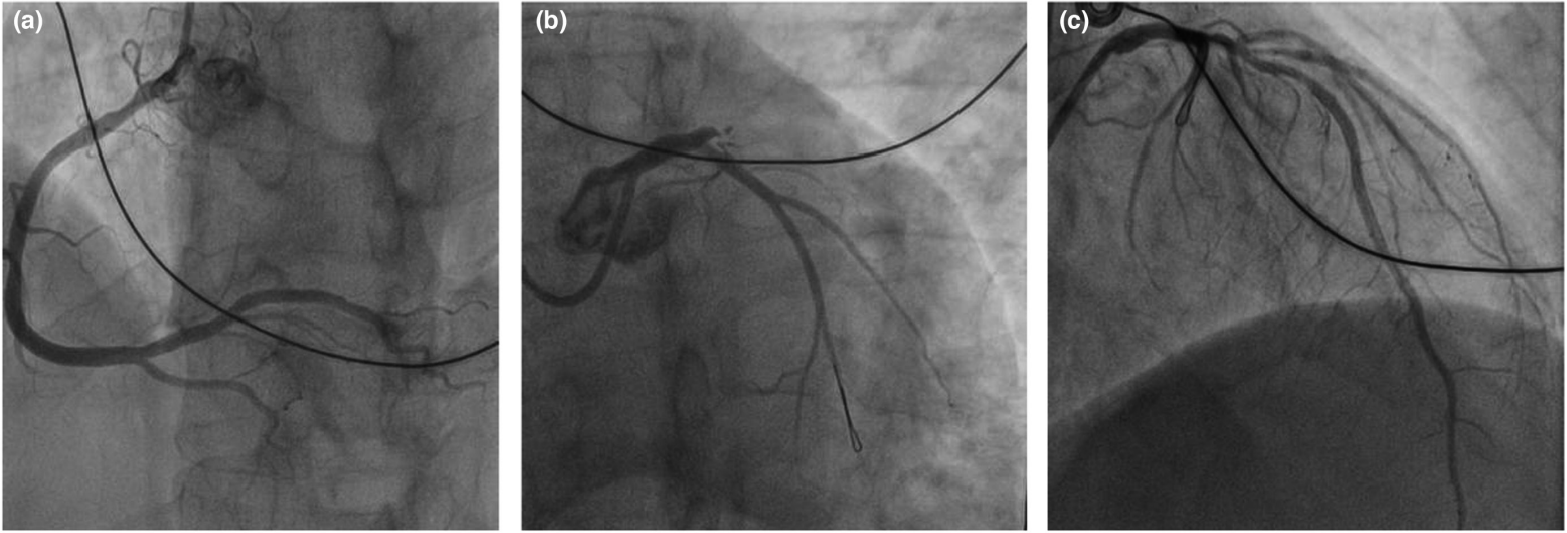
CAG demonstrating right coronary artery (a), occlusion of the proximal LAD (b), and an image of left coronary artery after PCI (c)

**FIGURE 4:**
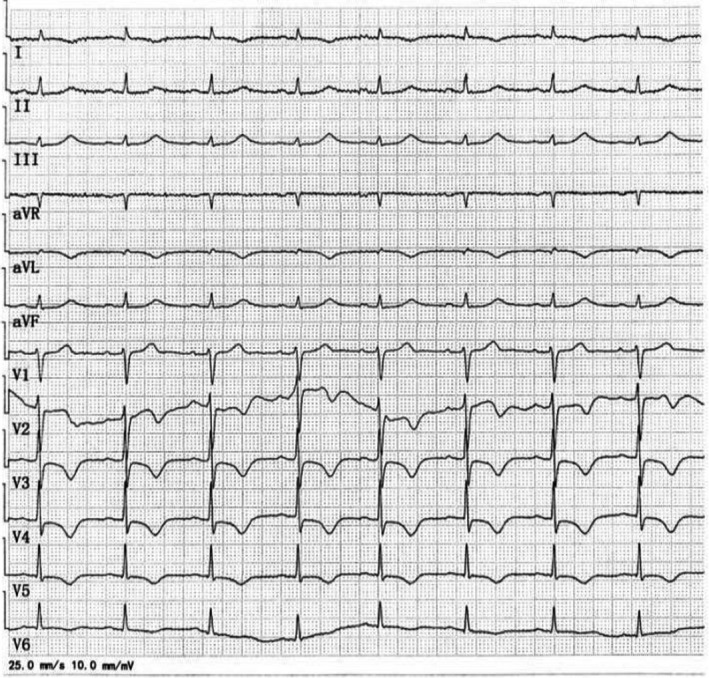
ECG on the first day after PCI presenting the resolution of right bundle branch block (RBBB), left anterior hemiblock (LAH), and left septal fascicular block

Based on the first two consecutive ECGs (Figures 1 and 2), what was the diagnosis?

The initial ECG (Figure 1) shows atrial fibrillation, left anterior hemiblock (LAH), ST‐segment depression in leads II, III, aVF, V_2_‐V_6_, and elevation in lead aVR (QTc: 403ms). These findings indicate the existence of non‐ST‐elevation myocardial infarction (NSTEMI). Notably, in the absence of right bundle branch block (RBBB), the increased amplitude of R waves in leads V_1_, V_2_ (R/S > 1 in V_2_), as well as the disappearance of “septal Q waves” in leads V_5_, V_6_, demonstrate the presence of left septal fascicular block. The second ECG (Figure 2) reveals sinus rhythm (obtained by electrical conversion), ST‐segment elevation in leads I, aVL and V_2_‐V_6_, depression in the inferior leads, V_1_ and aVR, accompanied with RBBB and LAH. These findings suggest the existence of ST‐elevation myocardial infarction (STEMI). In addition, there is a marked alternation in the amplitude of ST‐segment depression in lead V_1_, as well as the amplitude of ST‐segment elevation in leads V_2_‐V_6_ (indicated by the arrows). The QTc is extended to 532 ms.

In 2008, de Winter et al. (2008) found that in a small number of patients, when the proximal LAD, as a culprit artery, presented either subtotal occlusion or total occlusion with well‐developed collateral circulation, the ECG showed NSTEMI pattern with ST‐segment showing a 1‐ to 3‐mm upsloping depression at the J point in leads V_1_ to V_6_, followed by tall, positive symmetrical T waves and with 1‐ to 2‐mm ST‐elevation in aVR. This ECG pattern is now referred to as de Winter pattern in which the ST‐segment usually slightly depresses in inferior leads. In the present case, the first ECG was consistent with de Winter pattern and resulted from the proximal LAD lesion. According to the comparison between the ECG in Figure 1 and that in Figure 4, the left septal fascicular block was transient and was also caused by proximal LAD occlusion. In 2012, Fiol et al. (2012) first described a special ECG pattern, characterized by ST‐segment elevation in leads I, aVL, V_2_ to V_6,_ as well as ST‐segment depression in the inferior leads, and often with RBBB and LAH but without ST‐segment elevation in V_1_. This pattern occurs in patients with acute total occlusion of the left main trunk (LMT) without collateral circulation. In this situation, the left circumflex (LCX) coronary artery is involved, which attenuates ST‐segment elevation in leads V_1_ and aVR that should be generated by proximal LAD occlusion.

Coronary angiography revealed no collateral circulation in this patient, so the initial ECG could establish a diagnosis of de Winter pattern with acute subtotal occlusion of the proximal LAD. The second ECG demonstrated STEMI similar to that reported by Fiol et al. As shown by the second ECG, the ST‐segments of V_1_ and aVR were significantly depressed, further confirming that the LCX ostium was involved. At the same time, it is rare that the ST‐segment depression in V_1_ and the ST‐segment elevation in V_2_‐V_6_ exhibit beat‐to‐beat alternation. The evident electrical alternans reflects spatiotemporal heterogeneity in myocardial repolarization, predicting a higher risk of malignant ventricular arrhythmias (Narayan, 2006).

Although CAG showed total occlusion of proximal LAD, the ECG results indicated that the patient had experienced subtotal occlusion of proximal LAD to total occlusion of the LMT. The reason should be the thrombus increases rapidly within a short time. The lesson from this patient is that de Winter pattern is usually static and persisting, but sometimes, it could change into STEMI pattern associated with occluded proximal LAD and even occluded LMT. All of them are prone to cardiogenic shock and/or cardiac arrest. Therefore, it is vital to identify de Winter pattern accurately and perform PCI timely in view of the potential circulatory collapse, fatal arrhythmias, and sudden cardiac death from it.

The second ECG indicated total occlusion of the LMT, but only proximal LAD occlusion without LCX obstruction was shown in CAG. It may be explained by partial thrombus dissolution due to anti‐platelet aggregation and anticoagulation treatments, or mechanical factors associated with multiple defibrillation that had led to the displacement of the thrombus from LMT near the LCX ostium deeply into the proximal LAD.

The large, dominant right coronary artery, restoring blood flow in the LCX, and timely PCI may contribute to the patient's survival and favorable prognosis.

## CONFLICT OF INTEREST

The authors declare no conflicts of interest.

## ETHICAL APPROVAL

The study was approved by the Institutional Review Board Committee of The Third Affiliated Hospital of Soochow University, Changzhou City, China.

## AUTHOR CONTRIBUTIONS

Dr Xiaoqing Wang was responsible for the design and writing of the article. Dr Yongjun Chen and Dr Xiaoyu Yang performed the CAG and PCI on the patient. Dr Ling Yang was responsible for reviewing the article.

## Data Availability

No data are available.
